# Editorial: Molecular signalling and pathways contributing to neurodevelopmental disorders: insights into potential therapeutic avenues

**DOI:** 10.3389/fnmol.2023.1338013

**Published:** 2023-12-07

**Authors:** A. Kimberley McAllister, Deepak P. Srivastava, M. Dolores Martín-de-Saavedra

**Affiliations:** ^1^Center for Neuroscience, University of California, Davis, Davis, CA, United States; ^2^Department of Basic and Clinical Neuroscience, Institute of Psychiatry, Psychology and Neuroscience, King's College London, London, United Kingdom; ^3^MRC Centre for Neurodevelopmental Disorders, King's College London, London, United Kingdom; ^4^Department of Biochemistry and Molecular Biology, School of Pharmacy, Instituto Universitario de Investigación en Neuroquímica, Universidad Complutense de Madrid, Madrid, Spain

**Keywords:** neurodevelopmental conditions (NDCs), molecular mechanisms, therapeutic interventions, signaling pathways, genetics

Over the last decade, there have been growing interest and important developments in the understanding of the mechanisms involved in the pathophysiology of neurodevelopmental conditions (NDCs). Many of these advances have come from the development of multi-omics approaches, human cellular models, and an increased appreciation of the contribution of different cell types that may contribute to the pathophysiology of NDCs. The identification of genetic variants that increase the likelihood of NDCs has led to the “many genes, common pathways” hypothesis—the idea that genes involved in the pathophysiology of NDCs converge on common molecular mechanisms or act synergistically, leading to the core molecular and cellular phenotypes. There are also increasing lines of evidence suggesting that environmental factors may also interact with the genetic landscape associated with NDCs, resulting in increased risk for these conditions. However, more research is needed to identify those common mechanisms, their genetic causes, and the influence of environmental agents, with the final aim of developing therapeutic strategies to treat and/or prevent NDCs. In this Research Topic, five manuscripts describe new molecular signaling pathways contributing to NDCs and identify new potential therapeutic avenues.

Molloy et al. discuss the NRXN-NLGN-SHANK pathway, which is implicated in synaptic assembly, trans-synaptic signaling, and synaptic functioning. The review highlights the importance of studying synaptopathies in autism, providing an overview of the NRXN-NLGN-SHANK pathway in this context. The text also points to the high levels of variability in the approaches and models used to study the pathway and the lack of comparability across different models. The authors discuss the impact of this lack of reproducibility on the important goal of robust biomarker discovery for both identifying NDCs and measuring the efficacy of specific supports and/or treatments. The review also emphasizes the need for more neuroimaging studies to improve inferences about brain function and structural development in order to increase our understanding of the mechanisms underlying NDCs, including those in which there is a modification in the NRXN-NLGN-SHANK pathway.

Dai et al. discuss the use of multi-omics analysis to identify modified pathways in autism. The study identified 66 modified metabolites, while network analysis revealed that purine metabolism was one of the most strongly enriched pathways. Within the altered metabolites, uric acid was one of the most important. Interestingly, transcriptomic data showed differential expression of three purine metabolism-related genes (adenosine deaminase, adenylosuccinate lyase, and bifunctional enzyme neoformans 5-aminoimidazole-4-carboxamide ribonucleotide (AICAR) transformylase/inosine monophosphate (IMP) cyclohydrolase). The findings suggest that purinergic signaling may play a role in the etiology of autism. Further research is needed to corroborate the implication of this pathway and to understand whether uric acid could be used as a potential biomarker for autism.

Auvichayapat et al. discuss how transcranial direct current stimulation (tDCS) reduces the severity of specific characteristics in autistic individuals. This pilot study aimed to investigate the effects of tDCS on brain metabolite concentrations. Participants were male autistic children aged between 5 and 8 years. The study included a baseline evaluation, a treatment period of five consecutive days of tDCS, and a follow-up period of two weeks. The results showed that anodal tDCS led to changes in brain metabolite concentrations, indicating a modulation of brain function. The study also found associations between these changes and a 17% improvement in social functioning, as measured by the Autism Treatment Evaluation Checklist (ATEC). The authors found a significant association between decreased ATEC social subscale and N-acetylaspartate/creatine (Cr), choline/Cr, and myoinositol/Cr concentration changes in the locus coeruleus. These findings provide preliminary evidence for the potential of tDCS as a specific support for autism. As the authors mention in the manuscript, the study has limitations, including the small sample size and the lack of a control group, so further research is needed.

Hood et al. investigate the impact of phosphoinositide 3-kinases (PI3Ks) on neuronal development. PI3Ks are important enzymes involved in cell signaling and have been implicated in multiple cellular processes. Recent studies suggest that increased levels of the PI3KCD/p110δ isoform of PI3K may be relevant for conditions such as schizophrenia, autism, and intellectual delay. The authors investigated how increased p110δ expression impacts neuronal morphology, finding that it leads to decreased dendritic arborization and increased immature and mature dendritic spine densities. These findings provide insight into how increased p110δ expression may contribute to neurodevelopmental conditions.

Casanovas et al. dig into the isoform expression of the RNA Binding Fox-1 Homolog 1 (*RBFOX1*) gene in different brain regions. RBFOX1 is crucial for neuron development, and its decreased expression has been linked to NDCs. The authors identified multiple alternative *Rbfox1* transcript variants in the mouse cerebral cortex, including transcripts with novel first exons, alternatively spliced exons, and 3′-truncations. This research also shows that the different isoforms have stage- and region-specific expression in the mouse brain, implying that they play specific roles during brain development. This study provides insights into RBFOX1′s isoform diversity and regulatory mechanisms in the brain, which could help understand how its deletion impacts individuals with NDCs.

In conclusion, this Research Topic provides insights into the mechanisms regulating the signaling pathways contributing to NDCs and potential therapeutic interventions, including the NRXN-NLGN-SHANK pathway, purine metabolism, transcranial direct current stimulation, phosphoinositide 3-kinase p110δ, and RNA Binding Fox-1 Homolog 1.

## Author contributions

AM: Writing – review & editing. DS: Writing – review & editing. MM-d-S: Writing – original draft, Writing – review & editing.

